# Aberrant splicing of the hRasGRP4 transcript and decreased levels of this signaling protein in the peripheral blood mononuclear cells in a subset of patients with rheumatoid arthritis

**DOI:** 10.1186/ar3470

**Published:** 2011-09-20

**Authors:** Toko Hashimoto, Shinsuke Yasuda, Hideyuki Koide, Hiroshi Kataoka, Tetsuya Horita, Tatsuya Atsumi, Takao Koike

**Affiliations:** 1 Department of Medicine II, Hokkaido University Graduate School of Medicine, North 15, West 7, Kita-ku, Sapporo, 060-8638, Japan

## Abstract

**Introduction:**

An unidentified population of peripheral blood mononuclear cells (PBMCs) express Ras guanine nucleotide releasing protein 4 (RasGRP4). The aim of our study was to identify the cells in human blood that express hRasGRP4, and then to determine if hRasGRP4 was altered in any patient with rheumatoid arthritis (RA).

**Methods:**

Monocytes and T cells were purified from PBMCs of normal individuals, and were evaluated for their expression of RasGRP4 mRNA/protein. The levels of RasGRP4 transcripts were evaluated in the PBMCs from healthy volunteers and RA patients by real-time quantitative PCR. The nucleotide sequences of RasGRP4 cDNAs were also determined. RasGRP4 protein expression in PBMCs/monocytes was evaluated. Recombinant hRasGRP4 was expressed in mammalian cells.

**Results:**

Circulating CD14^+ ^cells in normal individuals were found to express hRasGRP4. The levels of the hRasGRP4 transcript were significantly higher in the PBMCs of our RA patients relative to healthy individuals. Sequence analysis of hRasGRP4 cDNAs from these PBMCs revealed 10 novel splice variants. Aberrantly spliced hRasGRP4 transcripts were more frequent in the RA patients than in normal individuals. The presence of one of these abnormal splice variants was linked to RA. The levels of hRasGRP4 protein in PBMCs tended to be lower. As expected, the defective transcripts led to altered and/or nonfunctional protein in terms of P44/42 mitogen-activated protein (MAP) kinase activation.

**Conclusions:**

The identification of defective isoforms of hRasGRP4 transcripts in the PBMCs of RA patients raises the possibility that dysregulated expression of hRasGRP4 in developing monocytes plays a pathogenic role in a subset of RA patients.

## Introduction

Ras guanine nucleotide releasing protein (RasGRP) 4 is a calcium-regulated guanine nucleotide exchange factor (GEF) and diacylglycerol (DAG)/phorbol ester receptor. The mouse, rat and human cDNAs and genes that encode this signaling protein were initially cloned during a search for novel transcripts selectively expressed in mast cells (MCs) by Yang and coworkers [1-3]. Others isolated a hRasGRP4 cDNA while searching for transcripts that encode oncogenic proteins in a patient with acute myeloid leukemia [4]. Mouse and human RasGRP4 mRNAs are abundant in an undefined population of peripheral blood mononuclear cells (PBMCs) [1,3]. Although all examined mature MCs in the tissues of normal humans and mice express RasGRP4 [1-3], it remains to be determined whether this signaling protein is expressed in another cell type.

Different isoforms of mouse, rat and human RasGRP4 [1,2,5] and its family member RasGRP1 have been identified which in each instance are caused by variable splicing of their precursor transcripts. For example, the *lag *mouse develops a lymphoproliferative disorder that resembles systemic lupus erythematosus (SLE) due to a failure to properly process the precursor mRasGRP1 transcript [6]. In support of these mouse data, we identified a subset of SLE patients that lacks the normal isoform of hRasGRP1 in their circulating T cells and PBMCs [7]. Splice variants of the hRasGRP4 transcript have been detected in the PBMCs of a limited number of patients with mastocytosis and asthma, as well as the HMC-1 cell line established from a patient with MC leukemia [1]. These data raised the possibility of altered expression of hRasGRP4 in some disease states.

RasGRP4 regulates the expression of many genes in the HMC-1 line, including the transcripts that encode prostaglandin D_2 _synthase, the transcription factor GATA-1, and the interleukin (IL)-13 inhibitory receptor IL13Rα2 [5,8]. In support of these *in vitro *data, the mature RasGRP4^+ ^MCs that reside in the peritoneal cavity of mice and rats preferentially metabolize arachidonic acid to prostaglandin D_2 _[9] due to their high levels prostaglandin D_2 _synthase [10].

Rheumatoid arthritis (RA) is a chronic inflammatory disease characterized by a distinctive synovitis resulting in progressive joint destruction. Although several genetic predispositions and environmental factors are known to increase the risk of developing RA, its pathogenesis is not completely understood [11,12]. MCs have been implicated in RA and experimental models of this autoimmune disorder. Tissue specimens isolated from the joints of RA patients contain increased numbers of hTryptase-β^+ ^MCs, and these effecter cells tend to localize at the junction of the pannus and cartilage, as well as in areas where the pannus is invading cortical bone [13-15]. MC-deficient WBB6F_1_-Kit^W^/Kit^W-v ^and WCB6F_1_-Kitl^Sl^/Kitl^Sl-d ^mice are resistant to arthritis induced by autoantibodies against collagen, glucose-6-phosphate isomerase, or methylated bovine serum albumin (BSA) [16-19]. Activated MCs produce a diverse array of proinflammatory factors, including varied granule serine proteases. In the K/BxN mouse serum-transfer [20] and methylated BSA/IL-1 [19] arthritis models, MC-restricted tryptase•heparin complexes regulate the accumulation of neutrophils and the loss of aggrecan proteoglycans in the cartilage.

MCs, monocytes, and macrophages originate from a common progenitor in humans [21], and hTryptase-β^+ ^MCs can be generated from human cord blood and PBMCs [22]. Circulating myeloid cells also differentiate into tissue-resident macrophage and dendritic cells. Macrophages are abundant in the RA synovium. Upon activation, these immune cells release substantial amounts of inflammatory cytokines and growth factors (for example, IL-1β, IL-6, tumor necrosis factor-α (TNF-α) and transforming growth factor-β) that participate in synovial inflammation and hyperplasia [23-25]. Thus, MCs and myeloid cells play pivotal roles in the pathophysiology of RA.

In the present study, we discovered that the CD14^+ ^myeloid cells in human PBMCs express hRasGRP4. As dysregulation of hRasGRP1 occurs in a subset of patients with SLE [7], we hypothesized that hRasGRP4 might be abnormally expressed in the PBMCs that give rise to MCs, macrophages, and possibly other cell types in some patients with RA. We now report that abnormal splicing of the hRasGRP4 transcript is frequent in the PBMCs of RA patients. The accumulated data raise the possibility that altered expression of hRasGRP4 occurs in a subset of RA patients.

## Materials and methods

### Healthy individuals and patients with RA and other autoimmune disorders

Forty-two apparently healthy Japanese individuals (6 males and 36 females, 49.8 ± 6.7 years old, mean ± SD) and 57 Japanese patients with RA (16 males and 41 females, 61.1 ± 13.5 years old, mean ± SD) were studied. All patients in the latter cohort were diagnosed as having RA by rheumatologists based on the American College of Rheumatology 1987 revised criteria for the classification of this autoimmune disease [26]. The mean disease duration of our RA patients was 126 months (range = 0 to 504 months). The Disease Activity Score in 28 joints (DAS28ESR4) [27] at the time of analysis was 3.3 ± 1.3 (range = 1.3 to 6.8). Fifty-one (89%) of these patients were receiving anti-rheumatic drugs. Thirty-seven (65%), 13 (27%), 7 (12%), and 39 (68%) of these patients were on methotrexate, sulphasalazine, bucillamine and prednisolone, respectively. Three patients were on biological agents. Thirty-six patients with other autoimmune diseases served as autoimmune controls. The patients in this control group had SLE (*n *= 10), polymyositis/dermatomyositis (*n *= 8), systemic sclerosis (*n *= 8), or the Sjögren's syndrome (*n *= 10). Our study was approved by the Human Ethics Committee of Hokkaido University Graduate School of Medicine, and informed consent was obtained from each subject.

### Cell separation

PBMCs were collected from approximately 10 ml of the peripheral blood drawn from healthy individuals or patients using Ficoll paque PLUS (Amersham Biosciences, Uppsala, Sweden). CD14^+ ^cells were purified from the resulting PBMCs using micro beads and a magnetic cell sorting separation unit (Miltenyi Biotec, Bergisch Gladbach, Germany). CD14, CD3, and CD19 micro beads were used to enrich non-monocyte, non-T cell, and non-B cells in the PBMCs by negative selection. This fraction is supposed to contain undifferentiated cells including mast cell progenitors [28]. T cells were also purified from the PBMCs using the RosetteSep human T -cell enrichment cocktail (StemCell Technology, Vancouver, BC, Canada). The purities of the obtained cells were routinely > 85% for CD14^+ ^myeloid cells and > 95% for CD3^+ ^T cells, as assessed on a FACS Calibur flow cytometer (BD Biosciences, San Jose, CA, USA) using phycoerythrin-labeled anti-CD14 and anti-CD3 antibody (BD Biosciences), respectively.

### Evaluation of hRasGRP4 transcript levels, and isolation of novel hRasGRP4 transcripts in RA patients

Total RNA was collected from whole PBMCs and separated cells using RNeasy Mini kits (Qiagen, Valencia, CA, USA). The obtained transcripts were converted into cDNAs employing QuantiTect Reverse Transcription kits (Qiagen). The coding regions of the hRasGRP4 cDNAs were then amplified by a PCR method using the forward 5'-AGCATGAACAGAAAAGACAGTAAG-3' and the reverse 5'-TGTCTAGGAATCCGGCTTGGA-3' primers which correspond to nucleotide sequences residing at the translation-initiation and -termination sites in the normal hRasGRP4 transcript noted at GenBank accession number [NM:170604], respectively. After a heat-denaturation step, each of the 25 cycles of the subsequent PCR steps consisted of a 15-s denaturing step at 94°C, a 30-s annealing step at 59°C, and a 1.5-minute extension step at 72°C. The transcript that encodes the housekeeping protein human glyceraldehyde-3-phosphate dehydrogenase (hGAPDH) served as a control in these transcript analyses.

A real-time quantitative PCR (qPCR) approach was used to monitor the overall levels of the hRasGRP4 transcripts in fractionated cell lineages and in PBMCs from 38 healthy individuals, 41 patients with RA, and 36 patients with other rheumatic diseases. In these experiments, the level of the hRasGRP4 transcript was normalized to that of the hGAPDH transcript using an ABI Prism 7000 Sequence Detection System (Applied Biosystems, Foster City, CA, USA) and TaqMan MGB probes specific for hRasGRP4 (Hs00364781m1) and hGAPDH (Hs00266705m1) (Applied Biosystems). We chose a hRasGRP4-specific primer set in these qPCRs that recognizes the junction nucleotide sequence located between exons 7 and 8. Relative quantification was performed using the comparable cycle threshold (C_T_) method in which ΔC_T _is the level of the hRasGRP4 transcript in the RNA sample relative to that of the hGAPDH transcript. The difference in the expression of the hRasGRP4 transcripts among each sample was defined as fold changes in mRNA levels by 2^-ΔΔCT^.

The nucleotide sequences of 295 hRasGRP4 transcripts were also determined using RNA isolated from 16 healthy individuals, 23 patients with RA (18 under treatment and 5 untreated), and 20 patients with other autoimmune diseases (5 with SLE, 5 with Sjögren's syndrome, 5 with inflammatory myositis and 5 with systemic sclerosis. In each instance, the generated hRasGRP4 cDNAs were subcloned into pcDNA3.1 V5-His-TOPO (Invitrogen, Carlsbad, CA, USA), and five arbitrarily selected cDNAs from each individual were sequenced using an ABI Prism 3130 Genetic Analyzer (Applied Biosystems).

### Evaluation of hRasGRP4 transcript levels in macrophages and osteoclasts

Macrophages were differentiated from peripheral blood CD14^+ ^cells in the presence of several cytokines using previously reported technology [29]. Briefly, macrophages were obtained by culturing CD14^+ ^cells in the presence of M-CSF (50 ng/ml). After seven days incubation at 37°C in a humid chamber, differentiated cells were collected. Osteoclasts were differentiated in the presence of M-CSF (33 ng/ml) and RANK-ligand (66 ng/ml) (Lonza Walkersville, Inc., Walkersville, MD, USA). After 14 days, cells were collected. RNA was collected from each cell lineage and hRasGRP4 expression was examined for both cell lineages using TaqMan MGB probes specific for hRasGRP4 and hGAPDH. Expression of cathepsin-K, one of the specific markers for differentiated osteoclasts, was evaluated for osteoclasts to confirm their differentiation (Probe ID: Hs00166156m1) (Applied Biosystems)[30]. RasGRP1 expression was also examined in the PBMC and in osteoclasts (Probe ID: Hs00996734m1) (Applied Biosystems).

### Use of an anti-peptide approach to obtain antibodies that recognize the N terminus of hRasGRP4

Rabbit anti-hRasGRP4 antibodies were generated against the novel 14-mer synthetic peptide MNRKDSKRKSHQEC that corresponds to the N terminus of the normal isoform of hRasGRP4. A Basic Local Alignment Search Tool (BLAST) protein search revealed no similar sequence in any other known human protein. Using this synthetic peptide, rabbit polyclonal anti-hRasGRP4 antibodies were generated and purified, as previously described for the generation of rabbit anti-hRasGRP1 antibodies [7]. The specificity of the generated anti-hRasGRP4 antibodies was confirmed by absorption assay using the same peptide as used for immunization both in immunoblot and in immunohistochemistry using lysates of epithelial cell line HEK-293 (line CRL-1573; American Type Culture Collection) transfected with expression constructs encoding hRasGRP4 with the C-terminal V5 epitope tag (data not shown).

### Generation of recombinant hRasGRP4 proteins using mammalian cell line and cell-free transcription-translation assay

Expression constructs encoding hRasGRP4 and its splice variants (variant 5 and 6) were transfected into the epithelial cell line HEK-293 that normally lacks hRasGRP4. The cDNAs that encode normal RasGRP4 and its splice variants were subcloned into pcDNA3.1 V5-His-TOPO (Invitrogen). We made hRasGRP4 constructs with or without C-terminal V5 tag. Transfections were performed using Lipofectamine 2000 Reagent (Invitrogen). The presence of the RasGRP4 at the protein level was evaluated by a SDS-PAGE immunoblot and by immunohistochemistry. A cell-free transcription: translation assay was performed using the PROTEINscript II T7 kit (Ambion, Austen, TX, USA) according to the manufacturer's instruction. Constructs encoding full-length normal RasGRP4, splice variant 5 and splice variant 6 were subjected to the system and evaluated by immunoblotting.

### Immunohistochemistry

Immunohistochemistry was carried out on PBMC-derived CD14^+ ^myeloid cells and T cells, and hRasGRP4-expressing HEK293 cells. Non-transfected HEK293 cells were used as negative control. A total of 500,000 cells in each instance were placed on a glass slide using a Shandon Cytospin 4 Cytocentrifuge (Thermo Fisher Scientific Inc., Waltham, MA, USA). hRasGRP4^+ ^HEK293 cells were cultured on a Lab-Tek II Chamber Slide System (Nalge Nunc International, Rochester, NY, USA). The prepared slides were fixed and permeabilized with 4% paraformaldehyde and 0.2% saponin (eBioscience, San Diego, CA, USA). Endogenous peroxide was quenched using a 3% solution of hydrogen peroxide in absolute methanol; blocking was done with a 3% solution of BSA in phosphate-buffered saline. Immunohistochemistry was performed using our rabbit anti-hRasGRP4 antibodies (1 μg/ml) or rabbit anti-β-actin antiserum (Sigma-Aldrich, St. Louis, MO, USA) diluted 1:80, followed by the relevant biotinylated antibodies and peroxidase-conjugated streptavidin (Nichirei Biosciences, Tokyo, Japan). Irrelevant rabbit IgG served as another negative control for our anti-hRasGRP4 antibodies. An absorption staining procedure was performed using a cocktail mixture of our anti-hRasGRP4 antibodies (1 μg/ml) and synthetic hRasGRP4-derived peptide (100 ng/ml). The immunoreaction was visualized using a 0.6% hydrogen peroxide (Nichirei Biosciences) solution containing 3,3'-diaminobenzidine tetrahydrochloride (DAB). Nuclear staining was done with hematoxylin, and the resulting stained cells were examined by light microscopy.

### Immunoblotting

After conjugation of our anti-hRasGRP4 antibodies with horse-radish peroxidase using Lightning-link HRP conjugation kit (Innova Biosciences, Cambridge, UK), the levels of hRasGRP4 protein in CD14^+ ^peripheral blood cells were evaluated using an immunoblot approach, as previously described [7]. Densities of immune-reactive bands were measured using ImageJ software supported by NIH [31]. Anti-phospho-P44/42 mitogen-activated protein kinase (MAPK) (Erk1/2) antibodies and anti-pan-P44/42 MAPK antibodies were purchased from Cell Signaling Technologies (Beverly, MA, USA).

### Statistical analysis

The chi-square test or Fisher's exact test was used to compare the frequencies of the identified hRasGRP4 variants in our patient's PBMCs. To evaluate the expression of a specific hRasGRP4 isoform, we first defined the normal range of ΔΔ-CT value as the mean ± 2 SD of the healthy volunteers. The levels of the hRasGRP4 transcript in the RA patients were then quantitated. The expression of hRasGRP4 transcripts in control individuals and patients were compared using Fisher's exact test. The incidence of splice variants and expression levels of this gene were compared by using Mann-Whitney's U-test. In all of the statistical analyses, JMP version 9.0 software (SAS Institute Inc., Cary, NC, USA) was utilized.

## Results

### Identification of hRasGRP4 mRNA and protein isoforms in CD14^+ ^myeloid cells

**Figure 1:**
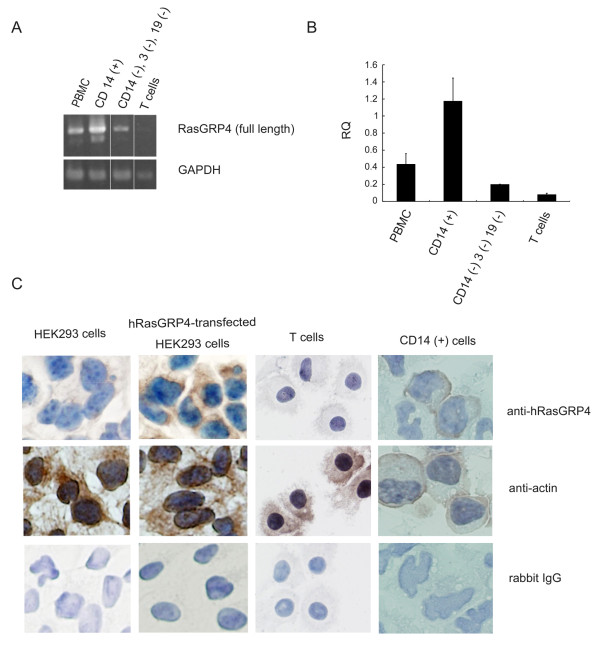
**Evaluation of hRasGRP4 expression in peripheral blood**. **A**, Evaluation of hRasGRP4 mRNA levels in unfractionated peripheral blood mononuclear cells (PBMCs), PBMC-derived CD14^+ ^cells, CD14^-^/CD3^-^/CD19^- ^cells and T cells isolated from healthy individuals. A RT-PCR/gel separation approach using an exon 1/exon 17 primer set in the hRasGRP4 gene was carried out to evaluate transcript expression in each cell type. Human glyceraldehyde-3-phosphate dehydrogenase (hGAPDH)-specific primers were used in the lower panels as positive controls. Representative results from three independent experiments are shown. **B**, A qPCR approach using a primer set that recognizes the junctional part of exon 7 and exon 8. Delta-C_T _of hRasGRP4 transcript level relative to the hGAPDH transcript in CD14^+ ^cells was defined as 1. qPCR assay was done in a triplicate manner for three times and the error bars indicate standard errors. **C**, Immunohistochemistry; PBMC-derived CD14^+ ^myeloid cells and T cells were stained with anti-hRasGRP4 antibody (top panels). For negative and positive controls, HEK293 cells that differed in their levels of hRasGRP4 protein also were stained with the anti-hRasGRP4 antibodies. For additional controls, replicate cells were stained with anti-β-actin antibodies (middle panels) or irrelevant rabbit IgG (bottom panels). Representative results from three to four procedures are shown.

Circulating *in vivo*-differentiated, unfractionated human PBMCs and PBMC-derived CD3^+ ^T cells, CD14^+ ^myeloid cells, and CD14^-^/CD3^-^/CD19^- ^cells were initially evaluated for the presence of hRasGRP4 mRNA using a semi-quantitative reverse transcriptase-PCR approach. Employing primers that correspond to the start and end of the protein's coding domain, the approximately 1.5-kb cDNA that encodes the normal isoform of hRasGRP4 was found to be abundant in the circulating CD14^+ ^cells present in the PBMCs of normal individuals (Figure 1A), as previously found [1]. The presence of large amounts of hRasGRP4 mRNA in these cells was confirmed by a real-time qPCR approach using different primers (Figure 1B). The non-T, non-B, non-monocyte population of CD14^-^/CD3^-^/CD19^- ^cells in these PBMCs contained relatively lower amounts of hRasGRP4 mRNA, and the level of the hRasGRP4 transcript was below detection in enriched peripheral blood T cells. In agreement with these transcript data, the CD14^+ ^cells purified from *in vivo*-differentiated human PBMCs contained hRasGRP4 protein as assessed immunohistochemically (Figure 1C). As expected, immunoreactive hRasGRP4 protein was not detected in T cells. Transfected HEK293 cells that differed in their levels of hRasGRP4 served as positive and negative controls.

### hRasGRP4 transcript levels during CD14^+ ^cell development into macrophages and osteoclasts

hRasGRP4 transcript levels decreased while CD14^+ ^peripheral blood cells differentiated into macrophages (Additional file 1/Figure s1A). RasGRP4 expression was diminished in osteoclasts (Additional file 1/Figure s1B, left panel), which was not countered at least by RasGRP1 (data not shown). Development of multi-nucleated osteoclasts was confirmed by light microscope. Elevated Cathepsin K expression was confirmed in these cells (Additional file 1/Figure s1B, right panel).

### Quantitative evaluation of hRasGRP4 transcripts in patients with RA and other autoimmune diseases

**Figure 2:**
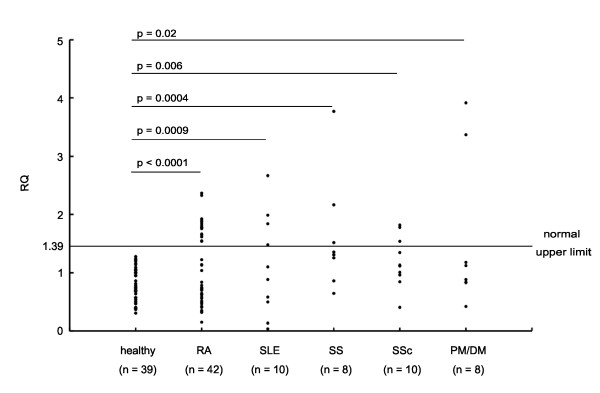
**Evaluation of hRasGRP4 mRNA levels in PBMCs**. A qPCR approach was used to quantify the overall levels of hRasGRP4 transcripts in the peripheral blood mononuclear cells (PBMCs) from healthy individuals and patients with rheumatoid arthritis (RA) and other rheumatic diseases such as SLE, Sjögren's syndrome (SS), systemic sclerosis (SSc), and polymyositis/dermatomyositis (PM/DM). Shown are the relative quantities (RQ) of human glyceraldehyde-3-phosphate dehydrogenase (hGAPDH)-corrected levels of the hRasGRP4 transcript in each sample. One of the healthy individuals was assigned to determine the value of 1. In all of the healthy individuals and patients, assays were done in a triplicate manner and mean values are plotted in the figure. Normal upper limit of the transcript level was defined as mean transcript level + 2SD of the healthy controls. Numbers of the patients whose hRasGRP4 transcript levels are above the normal upper limit were evaluated using Fisher's exact test.

We designated normal levels of hRasGRP4 transcripts in PBMCs as the mean ± 2 SD of that in the PBMCs of healthy individuals. The levels of the hRasGRP4 transcripts were higher than the normal levels in 41% of our RA patients (*P *< 0.0001) (Figure 2). The levels of the hRasGRP4 transcript also were higher in the PBMCs of patients that had other autoimmune diseases: SLE (*P *= 0.0009), polymyositis/dermatomyositis (*P *= 0.02), systemic sclerosis (*P *= 0.006), and Sjögren's syndrome (*P *= 0.0004). Thus, the presence of increased amounts of hRasGRP4 mRNA in PBMCs appears to be a useful marker for the identification of patients with autoimmune disorders. Despite these data, the levels of the hRasGRP4 transcript in the PBMCs of our RA patients was not correlated with the examined clinical features (for example, age, disease duration, DAS28, erythrocyte sedimentation rate (ESR), or serum matrix metalloproteinase 3 (MMP3)) (data not shown). Also in healthy individuals, RasGRP4 expression levels were not affected by age (data not shown). In addition, the levels of hRasGRP4 transcript were not affected by the ratios of monocytes in the PBMCs (represented by the sum number of lymphocytes and monocytes) from our RA patients (Additional file 2/Figure s2). Therefore, it would be acceptable for a screening to evaluate hRasGRP4 transcript levels using PBMC instead of using purified monocytes.

### Identification of 10 novel hRasGRP4 transcripts that have undergone defective splicing of the precursor transcript

**Figure 3:**
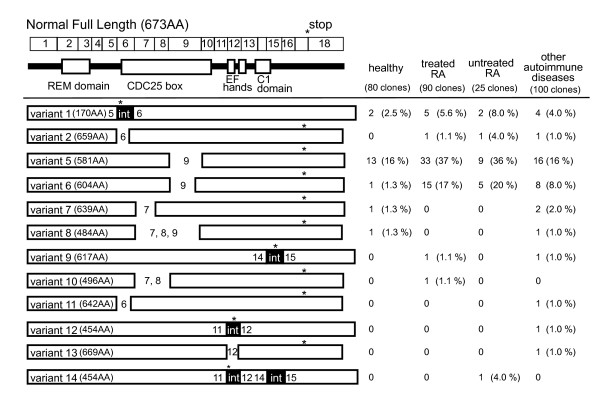
**Novel transcripts that encode abnormal hRasGRP4 isoforms in the PBMCs**. The top panel shows the exon structure and corresponding functional motifs of hRasGRP4, which include the Ras exchange motif (REM), CDC25-like GEF domain (CDC25 box), calcium-binding EF hands, and diacylglycerol -binding C1 domain. Splice variants 5 to 14 in the lower panel correspond to the 10 new hRasGRP4 transcripts identified in our RA patients. If translated, the number of amino acids (AA) in each expressed isoform is indicated. Eighty hRasGRP4-specific cDNAs were isolated and sequenced from 16 healthy individuals. Two, 13, 1, 1 and 1 of these cDNAs corresponded to splice variants 1, 5, 6, 7, and 8 respectively. The remaining 62 sequenced cDNAs in this control group corresponded to the normal, full-length hRasGRP4 isoform. Ninety and 25 hRasGRP4-specific cDNAs were isolated and sequenced from 18 RA patients undergoing treatment and from five untreated RA patients, respectively. The type and number of the identified defective transcripts in these patients are shown. One hundred hRasGRP4 cDNAs from 20 patients with other autoimmune diseases were sequenced, resulting in 36 defective and 64 normal hRasGRP4 transcripts as shown. Variants 1, 9, 12, and 14 possess a premature translation-termination codon (*). Intron 1 is 3.9 kb and many of the other introns in this approximately 17.2 kb human gene are > 1 kb. Because the PCR approach used to identify and quantitate the new isoforms would miss an isoform that contains intron 1, it is likely that additional splice variants exist in PBMCs that contain one or more large introns.

Sequence analysis of the hRasGRP4 cDNAs from 16 healthy individuals and 23 RA patients (including 5 patients on no therapy) revealed 12 isoforms of hRasGRP4 caused by alternative splicing of its precursor transcript (Figure 3). Four previously identified isoforms of hRasGRP4 have been designated as splice variants 1 to 4 [1]. Two of the alternative splicing isoforms identified in our RA patients correspond to variants 1 and 2. However, the other 10 isoforms (designated as variants 5 to14) have not been previously described. These novel splice variants that lack the entire exon 9 (splice variant 5, GenBank accession number: (FJ768677)); the first 207 nucleotides of exon 9 (splice variant 6, GenBank accession number: (FJ768678)); exon 7 (splice variant 7, GenBank accession number: (FJ768679)); exons 7, 8, and 9 (splice variant 8, GenBank accession number: (FJ768680]); exons 7 and 8 (splice variant 10, GenBank accession number: (FJ768682)); exon 6 (splice variant 11); and 12 nucleic acids at the 5' end of exon 12 (splice variant 13). Intron 14 had not been removed in splice variant 9 (GenBank accession number: (FJ768681)); 143 nucleotides from intron 11 had not been removed in splice variant 12; and 143 nucleotides from intron 11 and 95 nucleotides from intron 14 had not been removed from splice variant 14.

The most frequently found abnormal hRasGRP4 transcript identified in our group of RA patients was splice variant 5, which lacks the entire exon 9. Loss of this exon does not cause a frame-shift abnormality or a premature translation-termination codon in the processed transcript but does results in the loss of 92 amino acids which correspond to the C-terminal half of the CDC25-like catalytic domain in the signaling protein [1]. The second most frequent isoform was variant 6 which results in the loss of 69 of these same amino acids. Splice variants 9, 12, and 14 are more severely altered isoforms because they create in each instance a premature translation-termination codon. The hRasGRP4 splice variants as well as splice variant 6 were more frequent in clone number in the PBMCs of our RA patients (Table 1). Twenty clones corresponding to splice variant 6 from our RA patients were not from a few patients that express multiple clones of this variant. The distribution of splice variant 6 was one clone from eight RA patients and two clones from six RA patients. None of our RA patients had more than half of splice variant 6 from the sequenced clones. Except for splice variant 6, the frequencies of these splice variants were not significantly different in the PBMCs of patients with other autoimmune diseases relative to that of healthy subjects. Splice variant 6 was scarcely detected in the PBMCs of normal individuals. In healthy subjects, frequency of RasGRP4 splice variants was not related to their age (data not shown). In RA patients, the presence of splice variant 6 was not related to any evaluated clinical features (that is, age, disease activity, serum MMP3 levels, disease duration, or therapy treatment) (Table 2). However, this specific variant was more frequent in our male RA patients. The levels of hRasGRP4 transcripts evaluated by real-time qPCR were significantly high in individuals who possess splice variant 6 (*P *= 0.02, calculated using Mann-Whitney's U-test). Because abnormal splicing of RasGRP4 was most evident in patients with RA, we focused on RasGRP4 expression in RA patients in the following study.

**Table 1:** Statistical analysis of hRasGRP4 isoforms in healthy subjects and patients with RA and autoimmune controls

	normal	variant (%)	OR	95% CI	*P-*value
Any variant					
Healthy subjects	62	18 (22.5)			
RA patients (total)	47	74 (67.3)	5.42	2.86 to 10.3	0.00000016
RA patients (untreated)	9	16 (64.0)	6.12	2.32 to 16.2	0.00029
Autoimmune controls	69	31 (31.0)	1.55	0.79 to 3.04	0.27
	**non-variant 6**	**variant 6**	**OR**	**95% C.I**.	***P-*value**
Variant 6					
Healthy subjects	79	1 (1.3)			
RA patients	95	20 (18.0)	16.6	2.18 to 126	0.00035
Autoimmune controls	92	8 (8.0)	6.87	0.84 to 56.1	0.038

CI, confidential interval and *P-*values were calculated by Chi-square test to evaluate the incidence of alternative splicing with healthy subjects and patients groups. The frequency of variant 6 in each group was evaluated by Fisher's exact probability test. The percentage (%) of sequenced clones that contained an aberrant hRasGRP4 transcript is also indicated. OR, odds ratio; RA, rheumatoid arthritis

**Table 2:** Comparison of the clinical features between RA patients with hRasGRP4 variant 6 and those without

	variant 6 (-)	variant 6 (+)	*P*-value
Age (years)	62.6 ± 6.2	61.4 ± 11.2	0.96*
Male/female	0/10	6/7	0.012^†^
DAS28(ESR4)	3.8 ± 1.2	3.4 ± 1.85	0.31*
MMP-3	307 ± 127	148 ± 109	0.057
Disease duration (months) {mean (range)}	100 (0 to 492)	143 (0 to 504)	0.58*
Patients on therapy	4	8	
Patients on no therapy	2	3	0.77^†^

The *P*-values in the first four rows (*) were calculated using Mann-Whitney's U-test; the *P*-values (^†^) were calculated by chi-square test with Yates' correction. DAS28, The Disease Activity Score in 28 joints; MMP, matrix metalloproteinase; RA, rheumatoid arthritis

### hRasGRP4 protein levels in the PBMCs and CD14^+ ^peripheral blood cells isolated from healthy individuals and RA patients

**Figure 4:**
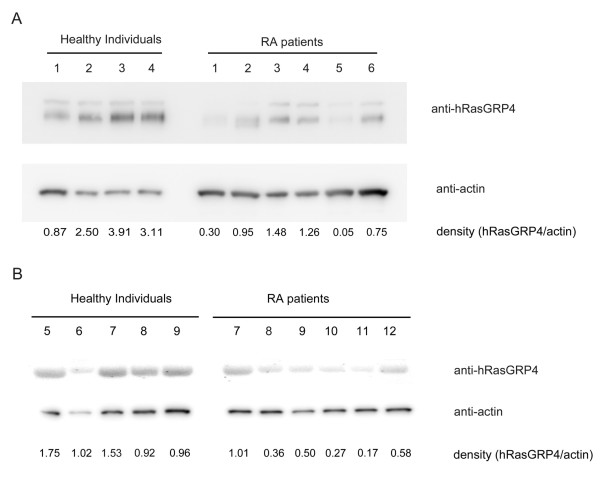
**Protein expression of hRasGRP4 in the peripheral blood evaluated by immunoblotting**. **A**. Immunoblotting of peripheral blood mononuclear cells from patients with RA and healthy controls. Lysates were prepared from peripheral blood mononuclear cells from patients with rheumatoid arthritis (RA) and healthy controls. Approximately 3 μg of protein from each lysate was subjected to SDS-PAGE. Immunoblotting was performed using peroxidase-conjugated anti-hRasGRP4 antibodies and anti-β-actin antibodies. PBMCs from four healthy individuals and six RA patients were evaluated in panels A. **B**. Immunoblotting of circulating CD14^+ ^cells isolated from five healthy controls and six RA patients.

The levels of hRasGRP4 protein were lower in the PBMCs from many of our RA patients relative to that of healthy control individuals (Figure 4A). Abnormal-sized bands corresponding to splice variant 5 or 6 were scarcely detected by our immunoblot analysis, except that patients 1 and 2 had detectable smaller-sized bands. Although there remains a possibility that our antibodies do not recognize alternatively-spliced isoforms, recombinant hRasGRP4 splice variant 6 with C-terminal V5 tag was recognized clearly by our anti-hRasGRP4 antibodies and by anti-V5 antibody (data not shown). Most of our RA patients express abnormal isoforms of the hRasGRP4 transcript from simultaneously obtained samples (Table 3). The levels of hRasGRP4 protein in CD14^+ ^peripheral blood cells were also lower in RA patients compared to those in healthy individuals (Figure 4B).

**Table 3:** Clinical profiles and sequenced hRasGRP4 cDNAs of the patients and healthy controls whose peripheral blood samples were obtained simultaneously as those in Figure 4A

	Age	Sex	DAS28	Treatment	Clone 1	Clone 2	Clone 3	Clone 4	Clone 5
**Healthy 1**	32	F	0	none	FL	FL	Variant 5	FL	FL
**Healthy 2**	31	F	0	none	FL	FL	Variant 5	Variant 6	FL
**Healthy 3**	38	F	0	none	FL	FL	Variant 7	FL	FL
**Healthy 4**	38	F	0	none	FL	FL	FL	FL	
**RA 1**	70	M	3.22	MTX 8 mg/week + IFX	FL	FL	FL	Variant 6	FL
**RA 2**	66	F	3.82	MTX 6 mg/week	FL	FL	FL	Variant 6	Variant 2
**RA 3**	61	F	2.17	MTX 10.5 mg/week + TAC 3 mg/day	Variant 5	Variant 5	Variant 5	Variant 5	Variant 5
**RA 4**	33	F	4.87	MTX 8 mg/week + PSL 5 mg/day	Variant 5	FL	Variant 5	FL	Variant 5
**RA 5**	78	M	2.65	MTX 6 mg/week + SSZ 1000 mg/day	FL	Variant6	Variant 5	Variant 5	Variant 6
**RA 6**	53	F	2.27	MTX 10 mg/week + PSL 2 mg/day	Variant 5	Variant 5	Variant 5	Variant 5	Variant 5

DAS28, The Disease Activity Score in 28 joints; FL, full-length; IFX, infliximab; MTX, methotrexate; PSL, prednisolone; RA, rheumatoid arthritis; SSZ, sulphasalazine; TAC, tacrolimus.

### Recombinant hRasGRP4 protein using cell-free transcription-translation assay and mammalian cell line

**Figure 5:**
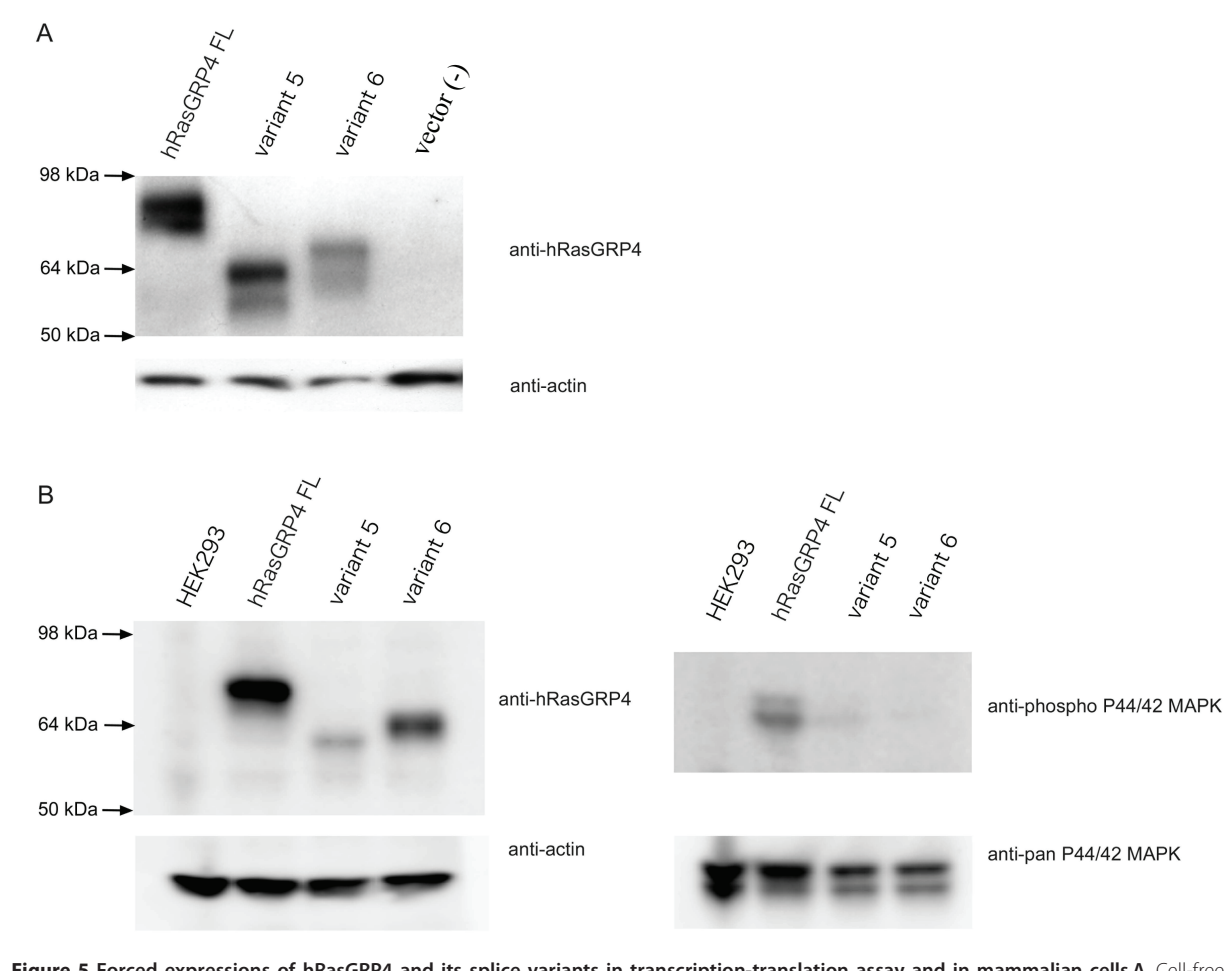
**Forced expressions of hRasGRP4 and its splice variants in transcription-translation assay and in mammalian cells**.**A**. Cell-free transcription translation assay. The vectors encoding full-length hRasGRP4, its splice variant 5 and 6 were subjected to cell-free transcription translation assay using rabbit reticulocyte lysate. Resulting products were subjected to immunoblotting using anti-hRasGRP4- horseradish peroxidase (HRP). **B**. Expression of hRasGRP4 and its splice variants in mammalian cells. The vectors encoding full-length hRasGRP4, its splice variant 5 or 6 were transfected into HEK293 cells. Cell lysates were subjected to immunoblotting using anti-hRasGRP4- HRP, anti-phospho or anti-pan P44/42 mitogen-activated protein kinase (MAPK) (Erk1/2). Representative results of three independent experiments are shown.

Full-length hRasGRP4, splice variant 5 and splice variant 6 were expressed at protein levels at expected sizes in a cell-free transcription-translation assay (Figure 5A). Similar protein expression was observed in mammalian cell-transfection system (Figure 5B, left panel). After transfection with full-length RasGRP4 into HEK293 cells, P44/42 MAP kinase activation naturally occurred, when compared to non-transfected cells (Figure 5B, right panel). Whereas, transfection with splice variant 6 barely activated P44/42 MAPK. Thus, as expected, hRasGRP4 splice variant 6 was functionally defective for the activation of Ras-Erk pathway.

## Discussion

As far as we know, this is the first report that hRasGRP4 is abundantly expressed in peripheral blood monocytes from healthy individuals both at mRNA and protein levels. This finding opens a new insight into the field of monocyte-lineage cell biology and of the diseases where this lineage cell plays a prominent role. In the latter part of the present study, we revealed dysregulation of hRasGRP4 in the PBMCs from patients with RA.

It has been concluded that the signaling protein RasGRP4 plays a prominent role in the final stages of development of mouse, rat, and human MCs [1,5,8]. Nevertheless, hRasGRP4 mRNA also has been detected in an undefined population of cells in mouse and human PBMCs [1]. In support of the latter data, the hRasGRP4 transcript also has been found in the transformed leukocytes isolated from a patient with acute myeloid leukemia [4]. Although MC-committed progenitors are present in the peripheral blood, these cells are rare in number and have a CD13^+^/CD14^-^/CD34^+^/CD117^+ ^phenotype [28,32]. We, therefore, speculated that another cell population might be responsible for the presence of large amounts of RasGRP4 mRNA and protein in normal mouse and human PBMCs. We now show that the CD14^+ ^myeloid cells in PBMCs express this GEF at the mRNA and protein levels (Figures 1A, C). Only 26 dbESTs of the approximately 8.3 million human dbESTs in the GenBank-UniGene database originated from the hRasGRP4 gene. Twelve of them are from blood or bone marrow, followed by five from the kidney. Thus, this signaling protein normally is highly restricted in humans. More than 300,000 dbESTs have been deposited in the database that originated from the adult human lung. Although the lung contains large numbers of macrophages, only one of the lung-derived dbESTs in the database originated from the hRasGRP4 gene. In support of these dbEST data, the levels of the RasGRP4 transcript in the mouse and human lung are below detection by RNA blot analysis [1]. Because hRasGRP4 mRNA and protein are below detection in macrophage-rich organs and because human PBMCs cease expressing hRasGRP4 when they are exposed to lectins *ex vivo *[1], we conclude that the monocytes in PBMCs cease expressing this signaling protein when they differentiate into mature macrophages in tissues. In agreement with this conclusion, *in vitro*-developed human macrophages and osteoclasts contained much lower hRasGRP4 mRNA levels when evaluated by qPCR (Additional file 1/Figure S1).

Because monocytic cell lineages are indispensable initiators/effectors in inflammatory arthritis, we next focused on hRasGRP4 expression in patients with RA, then evaluated hRasGRP4 expression in PBMCs from RA patients both quantitatively and qualitatively. In the present study, we note higher levels of hRasGRP4 mRNA (Figure 2) but also a higher frequency of certain defective hRasGRP4 isoforms in the PBMCs from RA patients (Figure 3 and Table 1). In our RA patients, the expression levels of hRasGRP4 were not related to any of investigated clinical and laboratory features. Alternatively spliced isoforms of hRasGRP4 have been reported in a patient with bronchial asthma, and were designated splice variants 1, 2, and 4 [1]. Although we also detected splice variants 1 and 2 transcripts in our cohort, the frequencies of these variants were low. Instead, we identified 10 novel splice variants including 2 major variants that are preferentially expressed in RA patients (Figure 3). The most abundant alternatively-spliced isoform was splice variant 5. This variant lacks exon 9 but the nucleotide sequence is kept in frame. The second abundant splice variant 6 lacks 5'-portion of exon 9 and also is in frame. This splice variant was scarcely found in healthy individuals, despite its relatively high prevalence in the patient group. In addition, splice variant 6 was related to high levels of hRasGRP4 mRNA as quantitated by qPCR. Because the probe used for qPCR theoretically recognizes the majority of the abnormal hRasGRP4 isoforms, such as splice variants 5, 6, 9, and 11 to 14, it is likely that cells which produce large amounts of defective splice variants attempt to compensate for that problem by producing more hRasGRP4 mRNA. In support of that conclusion, a naturally occurring mRasGRP4 splice variant was identified in the MCs developed from the C3H/HeJ mouse strain, which are unresponsive to phorbol esters [2]. In bone marrow-derived MCs developed from this mouse strain, the levels of the transcripts that encode this defective mRasGRP4 isoform were markedly higher relative to the corresponding MCs developed from A/J mice that preferentially express the normal isoform of mRasGRP4. The accumulated data suggest that when a certain lineage of cells is unable to produce a normal/functional signaling protein, such cells increase their production of defective transcripts in an attempt to compensate for the defective isoform. In fact, the peripheral blood cells from RA patients fail to express a substantial amount of normal hRasGRP4 protein (Figure 4A). Although splice variant 5 lacks the entire exon 9 and splice variant 6 uses an alternative splice donor site in exon 9, we did not find any point mutation in exon 9, splice donor site and splice acceptor site of this exon, even when the genomic DNA from a RA patient had exon 9 abnormality in all sequenced clones (data not shown). Although the reason why hRasGRP4 transcripts are defective in RA patients remains unclear, the presence of defective hRasGRP4 transcripts does not appear to be a treatment-induced phenomenon because non-treated RA patients also had a high frequency of defective hRasGRP4 cDNAs (Table 1). Other minor variants, such as splice variants 7, 8, and 11 which lack exons 1 to 3 and splice variant 13 which lacks the 5'-portion of exon 12, do not have any premature translation termination codon. Four of the other hRasGRP4 splice variants identified in our study comprise a premature translation-termination codon. Although these transcripts are candidates for nonsense-mediated mRNA decay, if translated, these splice variants would encode truncated non-functional hRasGRP4 isoforms that have lost their DAG/phorbol ester-binding sequence. How splicing of hRasGRP4 is controlled in monocytes remains to be determined in future studies. Functional aspects of alternative splicing of CD44 caused by its polymorphism have been implicated in rheumatoid arthritis [33-35]. Although we could not clarify genetic predispositions that are related to alternative splicing of RasGRP4, we suggest that full-length RasGRP4 protein levels are regulated, at least in part, by epigenetic factors such as alternative splicing.

Because exon 9 of hRasGRP4 encodes a large portion of the conserved catalytic CDC25 box in hRasGRP4, splice variants 5 and 6 are likely to be functionally defective if translated in CD14^+ ^cells *in vivo*. In fact, at least in our mammalian cell expression system, splice variant 6 was functionally defective in activating P44/42 MAPK when compared with normal hRasGRP4 (Figure 5B). Although the expression of splice variant 6 at the protein level in monocytes from RA patients was unclear, lower expression of RasGRP4 and/or that of functionally abnormal RasGRP4 isoform might affect the development of monocytes into macrophages or osteoclasts, resulting in altered function of these cells in RA patients.

## Conclusions

We clarified that hRasGRP4 is expressed in the CD14^+ ^monocytes in PBMCs. Because hRasGRP4 expression in monocytes is likely to be developmentally controlled, dysregulation of hRasGRP4 expression in peripheral blood monocytes may affect the cell functions of further differentiated cells such as macrophage and osteoclasts, playing pathologic roles in a subset of RA patients.

## Abbreviations

BLAST: Basic Local Alignment Search Tool; BSA: bovine serum albumin; DAB: 3,3'-diaminobenzidine tetrahydrochloride; DAS28: The Disease Activity Score in 28 joints; DAG: diacylglycerol; ESR: erythrocyte sedimentation rate; GEF: guanine nucleotide exchange factor; HRP: horseradish peroxidase; hGAPDH: human glyceraldehyde-3-phosphate dehydrogenase; IL: interleukin; MAPK: mitogen-activated protein kinase; MCs: mast cells; PM/DM: polymyositis/dermatomyositis; PBMCs: peripheral blood mononuclear cells; PBMCs: peripheral blood mononuclear cells; RasGRP: Ras guanine nucleotide releasing protein; RA: rheumatoid arthritis; REM: Ras exchange motif; RQ: relative quantities; SLE: systemic lupus erythematosus; SS: Sjögren's syndrome; SSc: systemic sclerosis; TNF-α: tumor necrosis factor-α

## Competing interests

The authors declare that they have no competing interests.

## Authors' contributions

TH designed and performed experiments and performed statistical analyses. SY designed the study, performed experiments and drafted the manuscript. HK designed and performed experiments, helped with collection and acquisition of the data and drafted the manuscript. HT helped with collection and acquisition of the data and drafted the manuscript. TA and TK were involved in the interpretation and design of the study, and also drafted the manuscript. All authors contributed to the final manuscript. All authors have read and approved the manuscript for publication.

## Supplementary Material

Additional file 1 **Figure S1. Evaluation of hRasGRP4 transcripts in CD14+ peripheral blood monocytes and *in vitro *cultured macrophages or osteoslasts**. **A**. hRasGRP4 transcripts in CD14+ peripheral blood monocytes and in-vitro cultured macrophages were evaluated using real-time qPCR. The level of hRasGRP4 transcripts against GAPDH in CD14+ cells at the first experiment was defined as 1. **B**. hRasGRP4 transcripts in in-vitro cultured osteoclasts were measured by real-time qPCR (left panel). Expression of cathepsin K (CTSK) was measured to confirm the development of osteoclasts (right panel). In these panels, amount of target gene transcripts against GAPDH transcripts in PBMC at one experiment were defined as 1. All experiments were done in a triplicate manner and error bars indicate standard errors. OC, osteoclasts; PBMC, peripheral blood mononuclear cells; RQ, relative quantification. Click here for file

Additional file 2 **Figure S2. hRasGRP4 transcript levels and the ratios of monocytes in the peripheral blood**. **A**. Relationship between hRasGRP4 transcript levels in PBMC from RA patients and the ratio of monocytes against the sum of monocytes plus lymphocytes. Linear relationship between Relative Quantification of hRasGRP4 in PBMC and the ratio of monocytes/(monocytes + lymphocytes) was measured using Spearman's rho analysis. **B**. Relationship between hRasGRP4 transcript levels in PBMC from RA patients and percentage of monocytes in the peripheral WBC. Linear relationship between Relative Quantification of hRasGRP4 in PBMC and the percentage of monocytes in WBC was measured using Spearman's rho analysis. PBMC, peripheral blood mononuclear cells; RQ, relative quantification. Click here for file
