# Supplementary figures and images for: Aberrant splicing of the hRasGRP4 transcript and decreased levels of this signaling protein in the peripheral blood mononuclear cells in a subset of patients with rheumatoid arthritis
Source: Arthritis Res Ther. 2011 Sep 20;13(5):R154. doi: 10.1186/ar3470 (PMC3308084; doi:10.1186/ar3470)

A

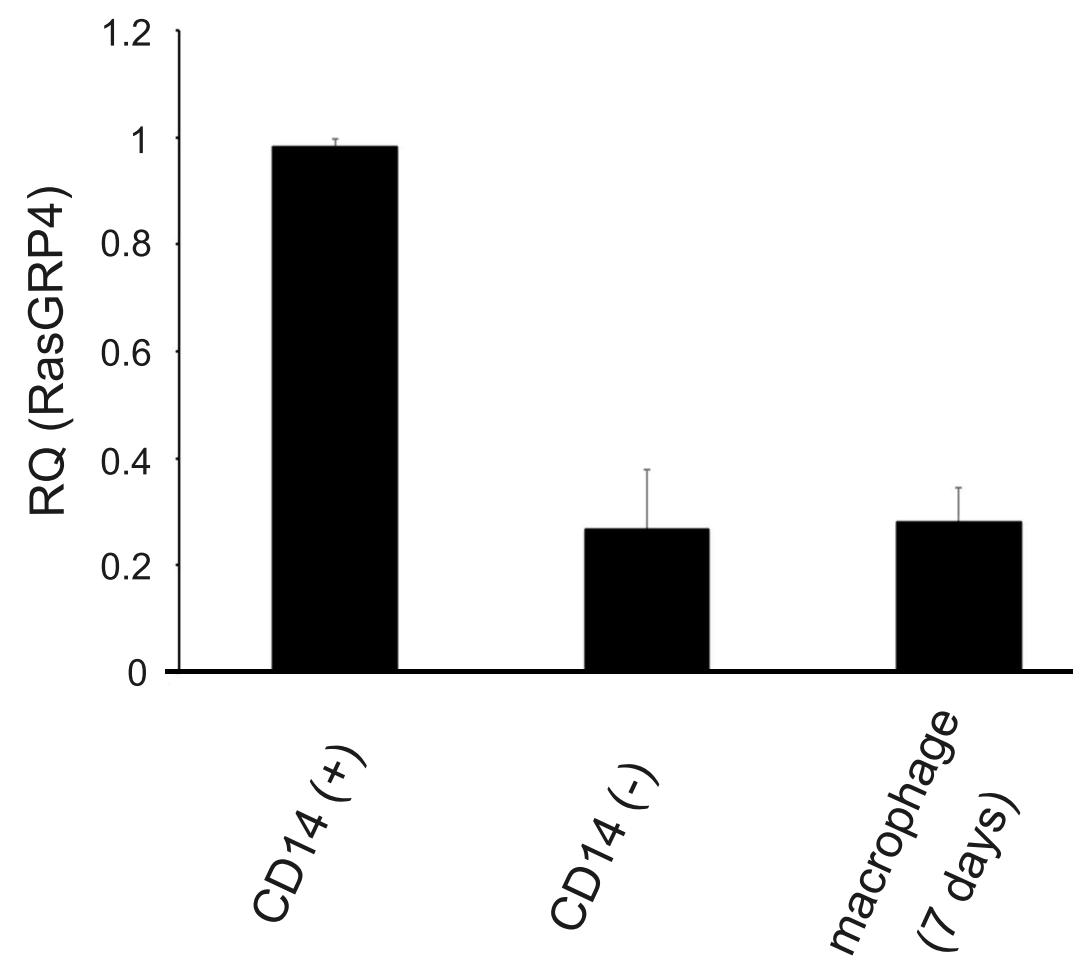

B

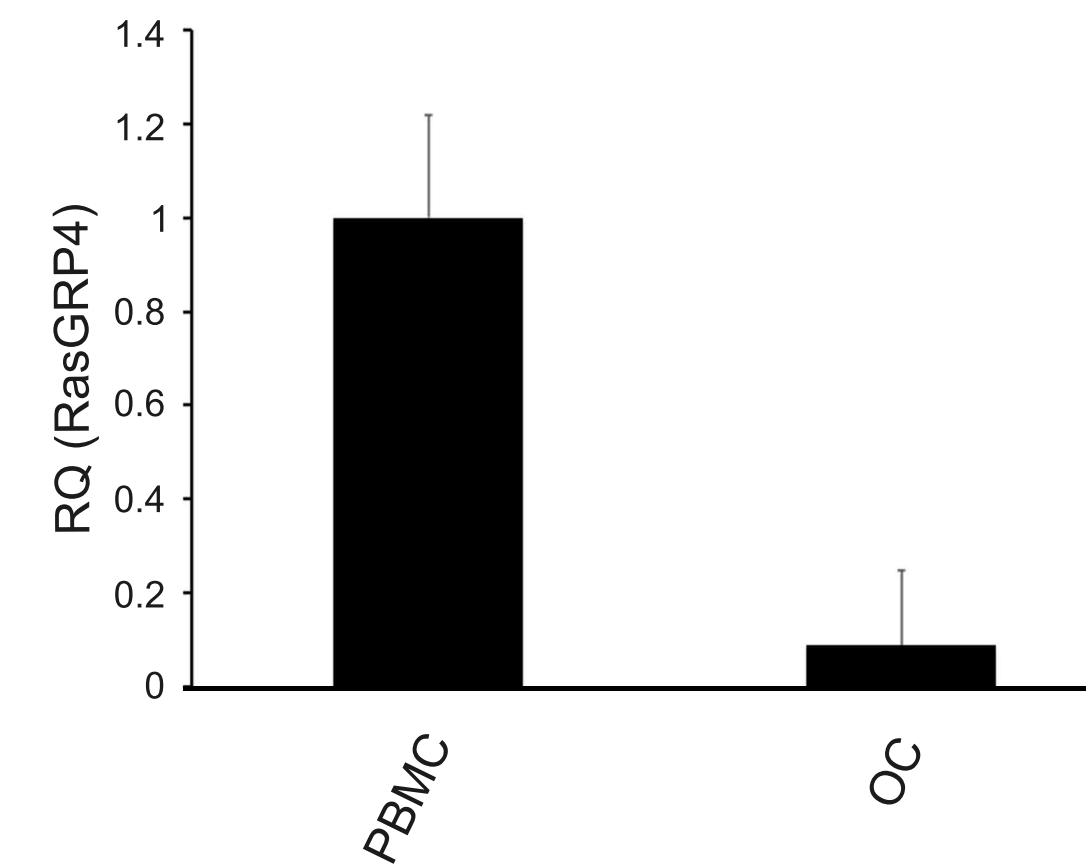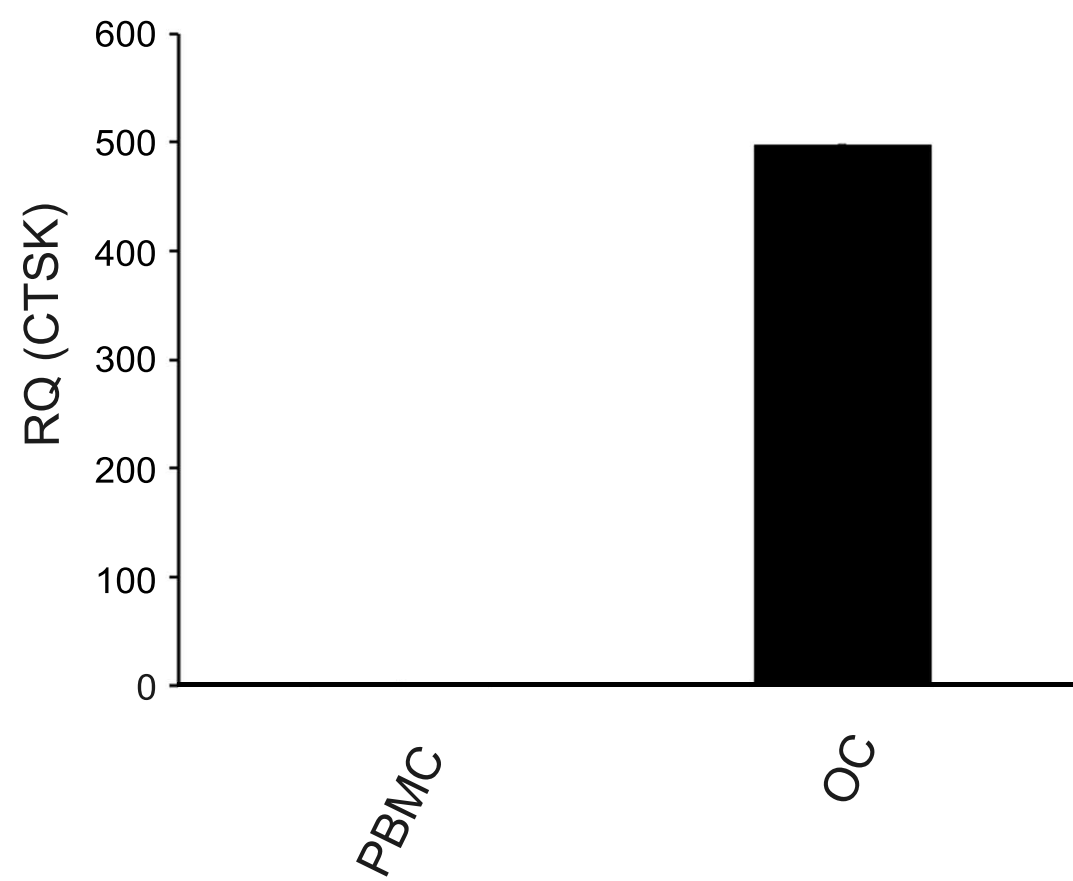

Supplement: Additional file 1 — Figure S1. Evaluation of hRasGRP4 transcripts in CD14+ peripheral blood monocytes and in vitro cultured macrophages or osteoslasts. A. hRasGRP4 transcripts in CD14+ peripheral blood monocytes and in-vitro cultured macrophages were evaluated using real-time qPCR. The level of hRasGRP4 transcripts against GAPDH in CD14+ cells at the first experiment was defined as 1. B. hRasGRP4 transcripts in in-vitro cultured osteoclasts were measured by real-time qPCR (left panel). Expression of cathepsin K (CTSK) was measured to confirm the development of osteoclasts (right panel). In these panels, amount of target gene transcripts against GAPDH transcripts in PBMC at one experiment were defined as 1. All experiments were done in a triplicate manner and error bars indicate standard errors. OC, osteoclasts; PBMC, peripheral blood mononuclear cells; RQ, relative quantification. [file ar3470-S1.PDF]

A

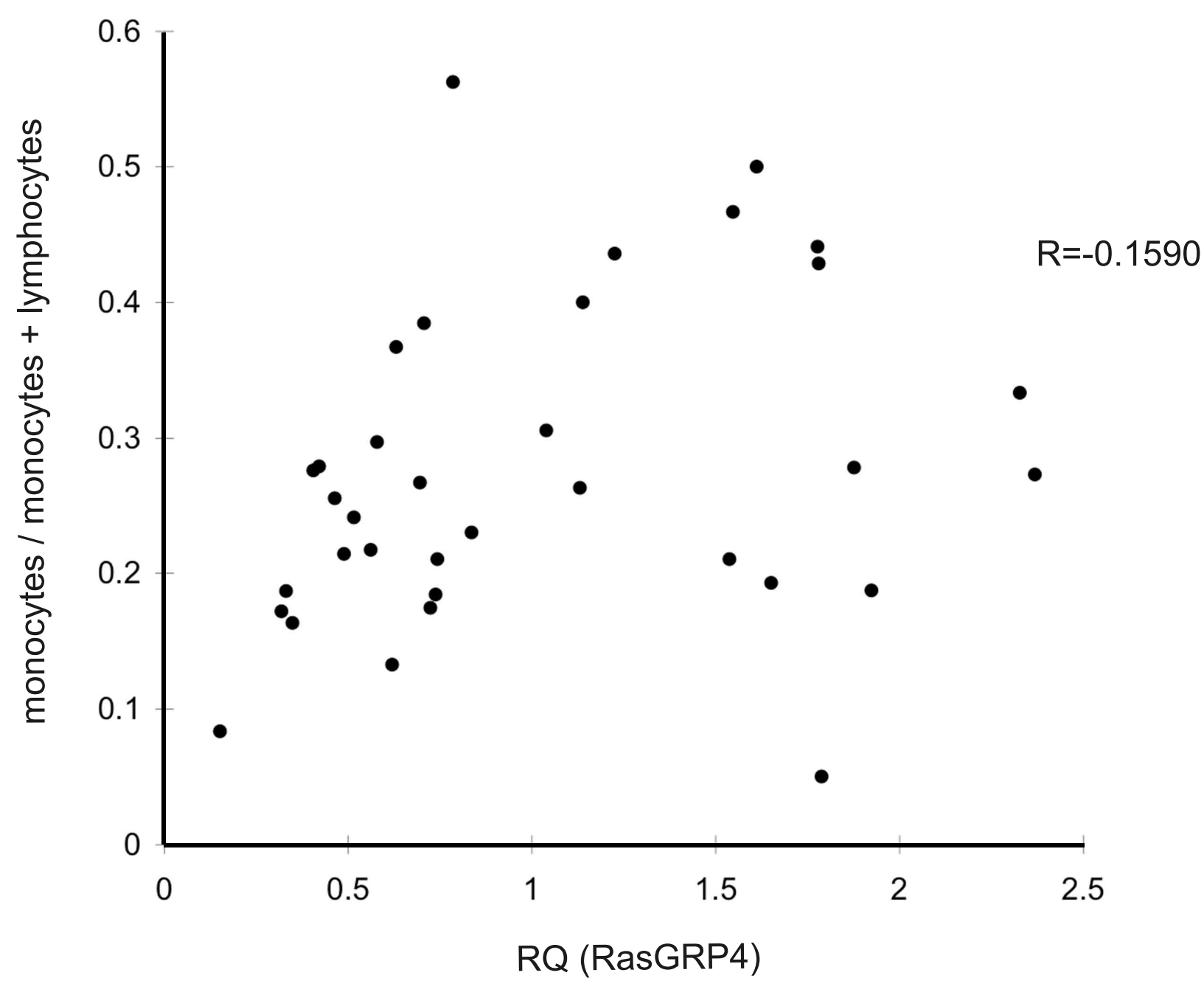

B

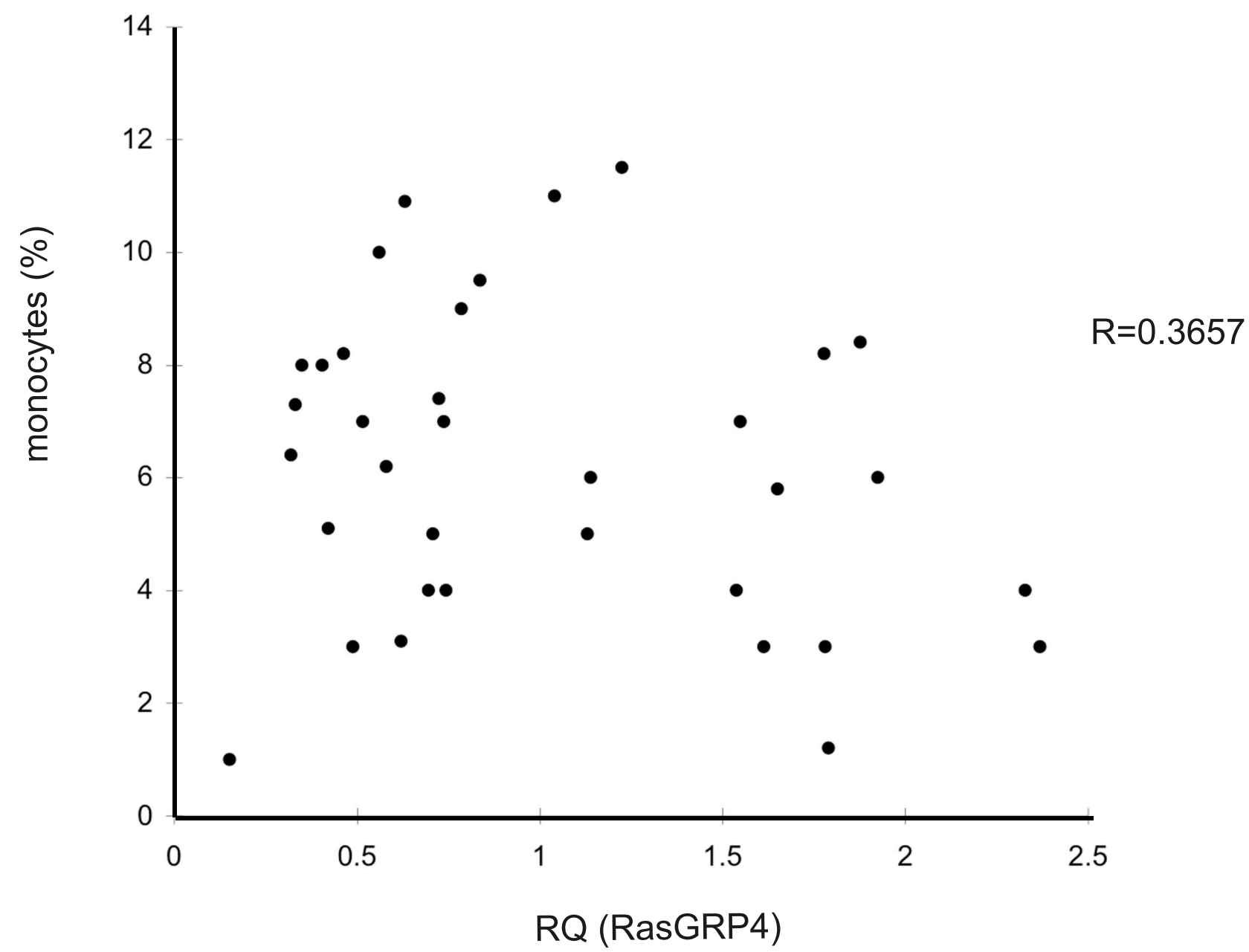

Supplement: Additional file 2 — Figure S2. hRasGRP4 transcript levels and the ratios of monocytes in the peripheral blood. A. Relationship between hRasGRP4 transcript levels in PBMC from RA patients and the ratio of monocytes against the sum of monocytes plus lymphocytes. Linear relationship between Relative Quantification of hRasGRP4 in PBMC and the ratio of monocytes/(monocytes + lymphocytes) was measured using Spearman's rho analysis. B. Relationship between hRasGRP4 transcript levels in PBMC from RA patients and percentage of monocytes in the peripheral WBC. Linear relationship between Relative Quantification of hRasGRP4 in PBMC and the percentage of monocytes in WBC was measured using Spearman's rho analysis. PBMC, peripheral blood mononuclear cells; RQ, relative quantification. [file ar3470-S2.PDF]
